# The Vasopressin Loading for Refractory septic shock (VALOR) study: a prospective observational study

**DOI:** 10.1186/s13054-023-04583-7

**Published:** 2023-07-21

**Authors:** Kensuke Nakamura, Hidehiko Nakano, Daisuke Ikechi, Masaki Mochizuki, Yuji Takahashi, Yasuaki Koyama, Hideki Hashimoto, Toshikazu Abe, Mineji Hayakawa, Kazuma Yamakawa

**Affiliations:** 1 Department of Critical Care Medicine, Yokohama City University Hospital, 3-9 Fukuura, Kanazawa-ku, Yokohama, Kanagawa 236-0004 Japan; 2 Department of Emergency and Critical Care Medicine, Hitachi General Hospital, 2-1-1, Jonan-cho, Hitachi, Ibaraki 317-0077 Japan; 3 Department of Emergency and Critical Care Medicine, Tsukuba Memorial Hospital, 1187-299, Kaname, Tsukuba, Ibaraki 300-2622 Japan; 4 Department of Health Services Research, Faculty of Medicine, University of Tsukuba, 1-1-1, Tennodai, Tsukuba, Ibaraki 305-8575 Japan; 5 Department of Emergency Medicine, Hokkaido University Hospital, Kita 14-jo Nishi 5-chome, Kita-ku, Sapporo-shi, Hokkaido 060-8648 Japan; 6 Department of Emergency Medicine, Osaka Medical College Hospital, 2-7, Daigakumachi, Takatsuki, Osaka 569-8686 Japan

**Keywords:** Critical care, Septic shock, Sepsis, Vasopressin, Loading, Bolus

## Abstract

**Background:**

Vasopressin is a second-line vasoactive agent for refractory septic shock. Vasopressin loading is not generally performed because of the lack of evidence for its effects and safety. However, based on our previous findings, we hypothesized it can predict the responsibility to vasopressin infusion with safety, and prospectively examined it in the present study.

**Methods:**

Vasopressin loading was performed via the intravenous administration of a bolus of 1 U, followed by its continuous infusion at 1U/h in patients with septic shock treated with ≥ 0.2 μg/kg/min noradrenaline. An arterial pressure wave analysis was conducted, and endocrinological tests were performed immediately prior to vasopressin loading. We classified patients into responders/non-responders based on mean arterial pressure (MAP) changes after vasopressin loading. Based on our previous findings, the lower tertile of MAP changes was selected as the cut-off. The change in the catecholamine index (CAI) after 6 h was assigned as the primary outcome. Digital ischemia, mesenteric ischemia, and myocardial ischemia during the admission period were prospectively and systematically recorded as adverse events.

**Results:**

Ninety-two patients were registered during the study period and examined. Sixty-two patients with a MAP change > 22 mmHg were assigned as responders and the others as non-responders. Blood adrenocorticotropic hormone levels were significantly higher in non-responders. Stroke volume variations were higher in responders before loading, while stroke volume and dP/dt_max_ were higher in responders after loading. Median CAI changes were − 10 in responders and 0 in non-responders, which was significantly lower in the former (p < 0.0001). AUROC of MAP change with vasopressin loading to predict CAI change < 0 after continuous infusion was 0.843 with sensitivity of 0.92 and specificity of 0.77. Ischemia events were observed in 5 cases (5.4%).

**Conclusions:**

Vasopressin loading may be safely introduced for septic shock. Vasopressin loading may be used to predict responses to its continuous infusion and select appropriate strategies to increase blood pressure.

**Supplementary Information:**

The online version contains supplementary material available at 10.1186/s13054-023-04583-7.

## Introduction

Vasopressin is one of the strongest vasopressor agents used to treat septic shock and acts via a different receptor from catecholamines 1. While randomized control trials previously demonstrated its efficacy [2–4, vasopressin causes adverse effects, such as ischemia events [5–7. Therefore, it is recommended as a second-line agent after noradrenaline to increase blood pressure 8.

Vasopressin is generally administered in a continuous infusion of up to 0.03 U/min (1.8 U/h) 8. However, continuous vasopressin administration takes some time to increase blood pressure from the decision to introduce vasopressin because the minimum concentration to increase blood pressure needs to be achieved 9, 10, although an urgent pressure increase is essential to treat septic shock refractory to noradrenaline. Furthermore, some patients respond poorly to vasopressin and, thus, other approaches need to be introduced immediately 11.

We previously examined vasopressin loading by intravenously administering a bolus of 1 U in 21 consecutive cases and retrospectively analyzed its effects 12. We demonstrated the potential of vasopressin loading to rapidly increase blood pressure and predict subsequent responses to its continuous infusion by identifying responders/non-responders to a bolus infusion without many adverse events. Based on these findings, we conducted a prospective observational study named the VAsopressin Loading for Refractory septic shock (VALOR) study, in which vasopressin loading was performed for patients with septic shock in whom vasopressin was required. We examined responses to bolus loading, assessed its safety, and clarified whether it may be used to predict responses to a subsequent continuous infusion using endocrinological and hemodynamic evaluations.

## Materials and methods

### Ethics approval and informed consent

This clinical study was conducted after receiving approval from the Ethics Board of our hospital (2020-130). It was registered at the University Hospital Medical Information Network (UMIN) with registration number UMIN000044041 13 (https://center6.umin.ac.jp/cgi-open-bin/ctr_e/ctr_view.cgi?recptno=R000050288). We received informed consent from eligible participants or their proxies.

### Patient selection

Patients admitted to the ICU at the Hitachi General Hospital Emergency and Critical Care Center between April 2021 and March 2023 were included. This medical and surgical ICU is reserved for patients from the emergency department, for patients exhibiting in-hospital acute deterioration, and severe patients following surgery. Inclusion criteria were as follows: (1) age ≥18 years, (2) septic shock (Sepsis-3 criteria), (3) requiring the administration of vasopressin following the continuous infusion of noradrenaline ≥0.2 μg/kg/min, (4) not given continuous steroid therapy until the administration of vasopressin, and (5) an arterial line inserted. Study exclusion criteria were designated as end of life/terminal care.

### Vasopressin administration protocol

Vasopressin was introduced to patients with septic shock who were administered noradrenaline ≥ 0.2 μg/kg/min. In the study protocol, vasopressin was intravenously administered as a bolus of 1 U for loading, followed by its continuous intravenous administration at 1 U/h. Prior to vasopressin loading, the following procedures were performed: (1) blood sampling for endocrinological tests and (2) ProAQT (Getinge, Japan) was connected to the arterial line for an arterial pressure wave analysis. Clinical practices were not limited other than vasopressin loading and its subsequent continuous infusion.

### Outcomes and measurements

The change in the catecholamine index CAI 6 h after vasopressin loading was assigned as the primary outcome 13. CAI was calculated with dopamine + dobutamine + (noradrenaline + adrenaline) × 100 μg/kg/min. The catecholamine dose was controlled independently from vasopressin loading by an Emergency and Critical Care physician to maintain the optimal blood pressure. The target of blood pressure was not specified by protocol and determined by each physician. The change in CAI ΔCAI 2 and 4 h after the introduction of vasopressin, urine output every 2 h after the introduction of vasopressin, 24 h fluid IN/OUT balance after vasopressin loading, lactate and pH changes for 2 h, the total vasopressin administration time, steroid use after the administration of vasopressin, in-hospital mortality, and the lengths of intensive care unit (ICU) and hospital stays were analyzed as secondary outcomes.

As reported in our previous study 12, we classified patients into responders and non-responders to vasopressin loading based on changes in mean arterial pressure (MAP) just after vasopressin loading. In this prospective study, the maximum MAP change was prospectively and carefully observed by the physicians within 3–5 min after loading, and was recorded as MAP change (ΔMAP). Since we previously selected a cut-off for a loading response from MAP change retrospectively obtained from medical records, we expected a different ΔMAP distribution and cut-off for this prospective study. Therefore, to follow our previous study in which the cut-off of 18 mmHg was the lower tertile, we herein selected the lower tertile of ΔMAP as the cut-off in this study.

We recorded hemodynamics, including an arterial pressure wave analysis, just before vasopressin loading and when maximum ΔMAP was achieved. Blood pressure from the arterial line, heart rate, cardiac output CO, stroke volume, stroke volume variation (SVV), central venous pressure, systemic vascular resistance SVR, and dPmx, the maximum pressure increase over time calculated in ProAQT, were evaluated. dPmx, generally expressed as dP/dt_max_, is expected to represent left ventricular contractility 14, 15, however, the limitation has been demonstrated that preload and actual aortic pressure also affected dP/dt_max_ 16. Digital ischemia, mesenteric ischemia, and myocardial ischemia during the admission period were prospectively and systematically recorded as adverse events.

Age, sex, height, body weight, acute physiology and chronic health evaluation (APACHE II) scores and sequential organ failure assessment (SOFA) scores on admission, comorbidities, and atrial fibrillation at the administration of vasopressin were analyzed as basic characteristics. The use of adjunctive therapies, such as mechanical ventilation, blood purification, and extracorporeal membrane oxygenation, was extracted. The timing of the administration of vasopressin from the initiation of noradrenaline, the noradrenaline dose and CAI when vasopressin was introduced, and maximum lactate levels within 24 h of the administration of vasopressin were also evaluated. In endocrinological tests, v, adrenocorticotropic hormone (ACTH), and cortisol levels were evaluated just before vasopressin loading.

### Sample size estimation

The primary outcome was already examined in 21 consecutive cases with significance in our previous retrospective study 12. Therefore, the sample size for the safety analysis was estimated in the present study. Previous studies reported ischemia events in 5–10% of cases without loading [2–4, 12]. When the adverse events ratio was assumed to be 5%, 73 patients were required to detect more than a + 5% increase with a 5% error. Therefore, we estimated that a maximum of 100 cases needed to be registered.

### Statistical analysis

The normality of the distribution of each parameter was assessed using the Shapiro–Wilk test. The significance of differences was evaluated using the Student’s *t*-test and chi-squared test for parametric data. The Mann–Whitney U test was performed for non-parametric data. The area under the receiver operating characteristic (AUROC) for ΔCAI 6 h < 0 was calculated with factors that significantly differed between responders and non-responders to vasopressin loading. For the sensitivity analysis, we performed the same analysis with the cut-off of 18 mmHg ΔMAP for responder/non-responder identification similarly with the previous study. All statistical analyses were conducted using JMP 14 software (SAS Institute Inc., Cary, NC, USA). Results were expressed as a mean ± standard deviation for normally distributed continuous variables with parametric test, or median (interquartile range) for non-normally distributed continuous variables with non-parametric test. *P*-values < 0.05 were considered to be significant.

## Results

**Fig. 1:**
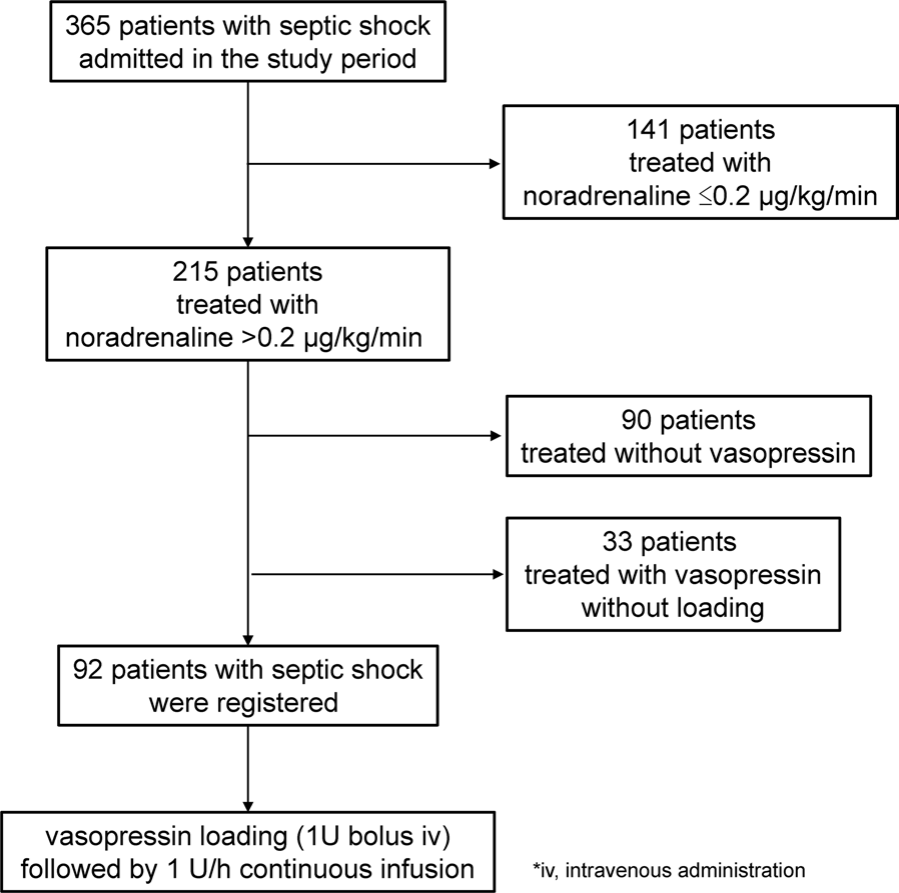
Study outline

A study outline is shown in Fig. 1. In the study period, 365 patients with septic shock were admitted into the ICU, and 215 were treated with ≥ 0.2 noradrenaline. Among them, 90 patients did not require the infusion of vasopressin and 33 for whom vasopressin loading was not performed were not registered to this study. Therefore, 92 patients were registered and administered a bolus of 1 U vasopressin followed by its continuous infusion at 1 U/h. The lower tertile of ΔMAP after vasopressin loading was 22 mmHg; therefore, 62 patients with ΔMAP > 22 mmHg were assigned as responders and 30 with ΔMAP ≤ 22 mmHg as non-responders.

Basic characteristics are shown in Table 1. Age, sex, height, weight, severity scores, adjunctive therapy, and atrial fibrillation did not significantly differ between responders and non-responders. The timing of the administration of vasopressin from the initiation of noradrenaline was longer in responders (5 (1.4, 15.7) vs. 1.75 (0.5, 4.9) h, p = 0.018), and blood lactate concentration within 24 h was lower in responders (2.6 (1.6, 4.6) vs. 4.7 (2.9, 8.1) mmol/l, p = 0.008), while the noradrenaline dose and CAI at the infusion of vasopressin did not significantly differ. In endocrinological tests, blood vasopressin and cortisol concentrations did not significantly differ between the groups; however, ACTH concentrations were significantly lower in responders (22.8 (9.7, 45.4) vs. 36.4 (22.3, 92.5) pg/ml, p = 0.018).

**Table 1:** Differences in baseline characteristics between responders and non-responders to vasopressin loading

n	Responders	Non-responders	p value
	MAP change > 22 mmHg	MAP change ≤ 22 mmHg	
	62	30	
Age, years	75.9 ± 11.6	77.7 ± 11.5	0.51
Male, n (%)	43 (69.4)	20 (66.7)	0.84
Height, cm	158.2 ± 8.4	159.9 ± 6.3	0.36
Body weight, kg	56.0 ± 13.8	52.0 ± 8.9	0.17
SOFA	8 (6, 10)	8 (5, 11)	0.93
APACHEII	19 (14, 23)	20 (13.5, 25.5)	0.99
Mechanical ventilation, n (%)	43 (69.4)	20 (66.7)	0.93
Blood purification, n (%)	22 (35.4)	14 (46.7)	0.22
Extracorporeal membrane oxygenation, n (%)	4 (6.5)	2 (6.7)	0.92
Atrial fibrillation n (%)	16 (25.8%)	6 (21.4%)	0.65
Vasopressin administration from noradrenaline start, hours	5 (1.4, 15.7)	1.75 (0.5, 4.9)	0.018
Noradrenaline dose before loading, μg/kg/min	0.3 (0.22, 0.48)	0.4 (0.3, 0.68)	0.14
Catecholamine index before loading	30 (22, 47.5)	47 (30, 67.5)	0.15
Max lactate within 24 h of loading, mmol/L	2.6 (1.6, 4.6)	4.7 (2.9, 8.1)	0.008
Vasopressin concentration, pg/ml	5.8 (3.5, 10.6)	6.7 (2.4, 19.8)	0.76
ACTH, pg/ml	22.8 (9.7, 45.4)	36.4 (22.3, 92.5)	0.018
cOrtisol, μg/dL	21.9 (14.6, 34.0)	26.1 (20.0, 41.2)	0.11

Data were expressed as a mean ± standard deviation for normally distributed continuous variables or median (interquartile range) for non-normally distributed continuous variables. Categorical variables were expressed as n (%)

*MAP* mean arterial pressure; *SOFA* sequential organ failure assessment; *APACHE* acute physiology and chronic health evaluation; *ACTH* adrenocorticotropic hormone

**Fig. 2:**
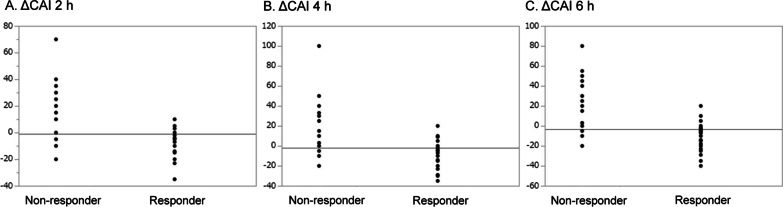
Changes in the catecholamine index in responders/non-responders to vasopressin loading. Responders to vasopressin loading were defined by a mean arterial pressure change > 22 mmHg after loading. Changes in the catecholamine index (CAI) (dopamine + dobutamine + (noradrenaline + adrenaline) × 100 μg/kg/min) from before loading to 2, 4, and 6 h after the initiation of vasopressin are shown. **A** Post 2 h—pre. **B** Post 4 h—pre. **C** Post—pre. Changes in CAI significantly differed between responders and non-responders at 2, 4, and 6 h

Outcomes are shown in Table 2 and Fig. 2. As the primary outcome, ΔCAI 6 h was − 10 in responders versus 0 in non-responders, which was significantly lower in the former (p < 0.0001). The AUROC of ΔMAP by vasopressin loading to predict ΔCAI 6 h < 0 was 0.843 (Additional file 1: Fig. S1). ΔMAP > 22 mmHg, the definition of responders in the present study, had sensitivity of 0.92 and specificity of 0.77 for ΔCAI 6 h < 0. Furthermore, ΔCAI 2 h and 4 h were significantly lower in responders (Fig. 2). This dataset did not have sufficient power to detect a statistically significant difference in in-hospital mortality between responders and non-responders (37.7% vs. 57.1%, p = 0.087). The length of hospital stay and duration of mechanical ventilation/blood purification were also not significantly different. Urine output 2, 4, and 6 h after the infusion of vasopressin was significantly higher in responders, and fluid volume IN and net IN–OUT balance were also significantly lower in responders. Blood lactate changes after 2 h were significantly lower with a minus value as the median in responders. The final duration of the administration of vasopressin was not significantly different between the groups, whereas the rate of steroid use after the introduction of vasopressin was higher in non-responders. As adverse effects, ischemia events were observed in 5 cases (5.4%). Digital ischemia occurred in 2 (7.1%) non-responders and 0 (0%) responders, which was significantly higher in the former (p = 0.030). No significant differences were noted in mesenteric ischemia or cardiac ischemia.

**Table 2:** Differences in outcomes between responders and non-responders to vasopressin loading

n	Responder	Non-responder	p value
	MAP change > 22 mmHg	MAP change ≤ 22 mmHg	
	62	30	
*Primary outcome*			
Catecholamine index change 6 h	− 10 (− 15, − 5)	0 (0, 28.8)	< 0.0001
*Secondary outcomes*			
Catecholamine index change 2 h	− 2 (− 10, 0)	0 (− 3.75, 20)	0.0005
Catecholamine index change 4 h	− 10 (− 12, 0)	0 (0, 22.5)	< 0.0001
In-hospital death, n (%)	23 (37.7)	16 (57.1)	0.087
Length of ICU stay, days	7 (5, 9)	6.5 (4.3, 11)	0.78
Length of hospital stay, days	21 (12, 47.5)	18.5 (6.25, 42.3)	0.44
Duration of mechanical ventilation, days	5 (4, 9)	6 (4.3, 10.3)	0.39
Duration of blood purification, days	5 (2.8, 19.3)	6(4.5, 12)	0.86
Urine output 0–2 h, ml/h	60 (20, 102.5)	20 (0, 70)	0.025
Urine output 2–4 h, ml/h	70 (20, 130)	50 (6.3, 92.5)	0.089
Urine output 4–6 h, ml/h	82.5 (35, 195)	42.5 (6.3, 107.5)	0.067
Fluid volume IN 24 h, ml	2217 (1525, 3052)	2809 (2026, 3483)	0.040
Fluid volume OUT 24 h, ml	1015 (674, 2460)	964 (331, 1756)	0.073
Net IN–OUT balance, ml	1007 (159, 1625)	1668 (966, 2736)	0.0064
Pre lactate, mmol/L	1.7 (1.3, 3.1)	2.4 (1.4, 4.7)	0.061
Post lactate, mmol/L	1.6 (1.2, 2.4)	2.8 (1.65, 5.45)	0.0009
Lactate change 2 h, mmol/L	− 0.2 (− 0.8, 0.1)	0.1 (− 0.38, 0.6)	0.0058
Pre pH	7.40 (7.32, 7.48)	7.38 (7.30, 7.44)	0.32
Post pH	7.41 (7.33, 7.46)	7.40 (7.29, 7.44)	0.48
Vasopressin administration time, hours	40 (26.5, 61.3)	45 (14.3, 85.9)	0.82
Steroid use after vasopressin administration, n (%)	15 (24.6)	16 (57.1)	0.0031
Digital ischemia, n (%)	0 (0)	2 (7.1)	0.030
Mesenteric ischemia, n (%)	1 (1.6)	1 (3.6)	0.58
Cardiac ischemia, n (%)	1 (1.6)	0 (0)	0.38

Data were expressed as a mean ± standard deviation for normally distributed continuous variables or median (interquartile range) for non-normally distributed continuous variables. Categorical variables were expressed as n (%)

*MAP* mean arterial pressure; *ICU* intensive care unit

Changes in hemodynamics are shown in Table 3. Prior to vasopressin loading, most of the parameters examined, including blood pressure, cardiac output, and central venous pressure did not significantly differ. Only SVV was higher in responders (17 (13, 22.3) versus 12 (7.8, 19.3) %, p = 0.015). After vasopressin loading, stroke volume and dPmx were significantly higher in responders: stroke volume 52 (42, 68) versus 42.5 (32.3, 54.5) ml, p = 0.025 and dPmx 1264 (1035, 1684) versus 956 (606.5, 1377), p = 0.024, respectively. However, the AUROC of SVV before loading and dPmx after loading for ΔCAI 6 h < 0 were low at 0.623 and 0.706, respectively. Similarly, the AUROC of ACTH for ΔCAI 6 h < 0 was low at 0.651. Although absolute value of CO and SVR were not different with significance, those changes before/after vasopressin loading were different significantly: CO change 0 (− 0.4, 0.5) versus − 0.4 (− 0.9, 0) l/min, p = 0.0034 and SVR change 569 (417, 858) versus 344 (159, 541) dynes/s/cm^5^, p = 0.0013, respectively.

**Table 3:** Differences in hemodynamics between responders and non-responders to vasopressin loading

n	Responder	Non-responder	p value
	MAP change > 22 mmHg	MAP change ≤ 22 mmHg	
	62	30	
Pre SBP mm Hg	107.3 ± 22.2	104.8 ± 22.2	0.59
Pre DBP mm Hg	51.0 ± 10.2	50.8 ± 11.4	0.59
Pre MAP mm Hg	68.6 ± 11.8	69.3 ± 15.1	0.84
Pre HR /min	93.6 ± 22.6	98.1 ± 22.6	0.38
Pre CO l/min	4.4 (3.5, 6.3)	4.2 (3.7, 5.3)	0.65
Pre SV ml	49 (40, 58)	42.5 (34.3, 63.5)	0.41
Pre SVV %	17 (13, 22.3)	12 (7.8, 19.3)	0.015
Pre dPmx	856 (658, 1190)	856 (576, 1284)	0.87
Pre SVR dynes/s/cm^5^	1162 (844, 1441)	1190 (845, 1413)	0.99
Pre CVP mm Hg	5 (5, 7.5)	5 (4, 9.3)	0.85
Post SBP mm Hg	157.8 ± 25.5	126.0 ± 28.2	< 0.0001
Post DBP mm Hg	75.3 ± 12.9	62.0 ± 14.8	< 0.0001
Post MAP mm Hg	105.2 ± 12.5	81.5 ± 17.3	< 0.0001
Post HR/min	88.6 ± 19.9	93.9 ± 22.6	0.27
Post CO l/min	4.6 (3.3, 5.7)	3.8 (2.8, 5.0)	0.11
Post SV ml	52 (42, 68)	42.5 (32.3, 54.5)	0.025
Post SVV %	13 (7.5, 17)	9.5 (8, 19.5)	0.94
Post dPmx	1264 (1035, 1684)	956 (606.5, 1377)	0.024
Post SVR dynes/s/cm^5^	1702 (1490, 2348)	1465 (1082, 1979)	0.068
Post CVP mm Hg	9 (5, 10)	7.5 (5.75, 12)	0.53

Data were expressed as a mean ± standard deviation for normally distributed continuous variables or median (interquartile range) for non-normally distributed continuous variables

*MAP* mean arterial pressure; *SBP* systolic blood pressure, *DBP* diastolic blood pressure; *HR* heart rate; *CO* cardiac output; *SV* stroke volume; *SVV* stroke volume variation, *SVR* systemic vascular resistance; *CVP* central venous pressure

The sensitivity analysis with the cut-off of 18 mmHg ΔMAP for responder/non-responder identification were shown in Additional file 2: Tables S1, S2 and S3. Similar results were obtained.

## Discussion

Responses to vasopressin loading may predict CAI changes after its continuous infusion with adequate sensitivity. Adverse events were not common following the administration of a bolus of 1 U vasopressin. Although responses correlated with some hemodynamics and endocrinological changes, they alone did not predict responses to the continuous infusion of vasopressin.

Rapid increases in blood pressure may be achieved with vasopressin loading because the maximum MAP change was observed within a few minutes in the present study. Since a continuous infusion requires more time for its administration to patients due to its preparation and priming/titration, vasopressive agents, including catecholamines, may need to be loaded in a bolus for emergency and critical care cases 17. Vasopressin increases blood pressure more slowly than catecholamines. Vasoconstriction and blood pressure increases induced by vasopressin are only achieved when plasma vasopressin concentrations are higher than 50 pg/ml 10. In contrast, noradrenaline and other catecholamines induce vasoconstriction linearly from the lowest concentration 18. These differences in the concentration-vasoconstriction relationship are due to the V1 and α1 receptors 19. Moreover, the half-life of vasopressin is 10–35 minutes 9, while that of catecholamines is a few minutes 20. Therefore, vasopressin loading appears to be appropriate for achieving a blood pressure target and steady state.

The bolus administration of vasopressin has not yet been examined in detail in clinical settings. Terlipressin, an analogue of vasopressin with a longer half-life 21, was administered with bolus loading in some clinical trials 22. Adverse events were only observed in patients in whom massive loading was performed 23. Regarding vasopressin, one small study reported 7 cases for which a bolus of 50 mU/kg vasopressin was administered, and 4 died due to mesenteric ischemia 24. In the present study, the frequency of adverse events was adequately low and similar to those reported in previous clinical trials on vasopressin without loading 3. The present results indicate that loading with a bolus dose of 1 U is safe.

It is important to predict responses to vasopressin because responses to its continuous infusion differ among patients. Although marked improvements occur in some patients 1, others do not respond to vasopressin 11, and, thus, other blood pressure management strategies need to be introduced. Strategy development based on responses to vasopressin loading is required, including a continuous infusion of vasopressin for responders and another intervention, such as the infusion of steroids or epinephrine, for non-responders without waiting to attempt the continuous infusion of vasopressin. Actually, it would be rather harmful that lactate was increased and cardiac output was decreased significantly in non-responders in this study. Randomized control trials to examine the effects of vasopressin loading with these strategy changes on the prognosis of refractory septic shock are warranted in the future.

In the present study, endocrinological testing immediately prior to vasopressin loading was performed to assess relationships with responses to vasopressin. Previous studies demonstrated that the concentration of vasopressin was elevated in early septic shock, but decreased within 48 h as shock continued 25, 26, which is referred to as vasopressin deficiency. While vasopressin deficiency did not correlate with responses to vasopressin, the timing of the administration of vasopressin from the shock onset was longer in responders in the present study. These findings indicated that responders might include the less sick patients, or that there might exist the vasopressin deficiency in which it could not be explained by the absolute vasopressin concentration at one point, such as hypovolemia. Meanwhile, ACTH concentrations were significantly higher in non-responders. Vasopressin is one of the feedback regulators of ACTH release via the pituitary vasopressin 3 receptor 27. Although vasopressin concentration was not different between the groups, vasopressin excretion might have been continued longer in non-responders. In these patients, relative adrenal insufficiency was suspected and steroid supplementation may be an alternative treatment. One of the reasons why some randomized control trials showed that the effectiveness of vasopressin was maximized with steroid use 11 may be that steroids acted as a rescue therapy for vasopressin non-responders.

SVV, the dynamic index for fluid responsibility, was higher in responders. Since vasopressin induces venous vessel contraction 9, it may have increased venous return by contracting stressed volume. dP/dt_max_ is a marker for cardiac contractility, when the appropriate preload and actual aortic pressure were maintained 15, 16. Vasopressin theoretically does not affect cardiac contractility, however, it would achieve a favorable hemodynamic adjustment with vaso-constriction, which resulted in dP/dt_max_ increase. If the cardiac contractility was originally better, vasopressin would be more effective, corresponding to our results.

These significant endocrinological and hemodynamics differences would be reasonable for vasopressin responses, but did not adequately predict the responses. Responses to vasopressin may be affected by many factors other than hemodynamics and endocrinological changes; therefore, vasopressin loading may be important for predicting these responses.

Furthermore, vasopressin loading increased urine output in a short period in responders. Because vasopressin increased vascular resistance more in efferent glomerular arterioles than in afferent glomerular arterioles 28, it was demonstrated that vasopressin could have increased urine output in early phase comparing with noradrenaline in septic shock 1. As the urine output was significantly different between responders and non-responders, vasopressin loading might have the predictability of renal impacts in septic shock.

There are several limitations that need to be addressed. This was an observational study. Therefore, a randomized control trial is needed to confirm the effects of vasopressin loading. Furthermore, the management of CAI was performed by an Emergency and Critical Care physician, who was not always available to make rapid adjustments to CAI in ICU patients. In addition, since Japanese ICU patients are often smaller and older than those in Western countries, a dose of 0.03 U/min (1.8 U/h) may be too high; therefore, we adopted the described protocol.

## Conclusions

Vasopressin loading may be introduced for septic shock to safely achieve a rapid increase in blood pressure. Vasopressin loading may be used to predict responses to its continuous infusion and select appropriate strategies to increase blood pressure.

### Supplementary Information

**Additional file 1**. **Fig. S1**. Receiver operatorating characteristic curve of mean arterial pressure change by vasopressin loading to predict catecholamine index change after 6 h < 0.

**Additional file 2**. **Tables S1, S2 and S3**. The sensitivity analysis of the baseline, outcomes and hemodynamics with the cut-off of 18 mmHg ΔMAP for responder/non-responder identification.

## Data Availability

The datasets generated and analyzed during the present study are available from the corresponding author upon reasonable request.
