# Supplementary figures and images for: The Vasopressin Loading for Refractory septic shock (VALOR) study: a prospective observational study
Source: Crit Care. 2023 Jul 21;27:294. doi: 10.1186/s13054-023-04583-7 (PMC10362561; doi:10.1186/s13054-023-04583-7)

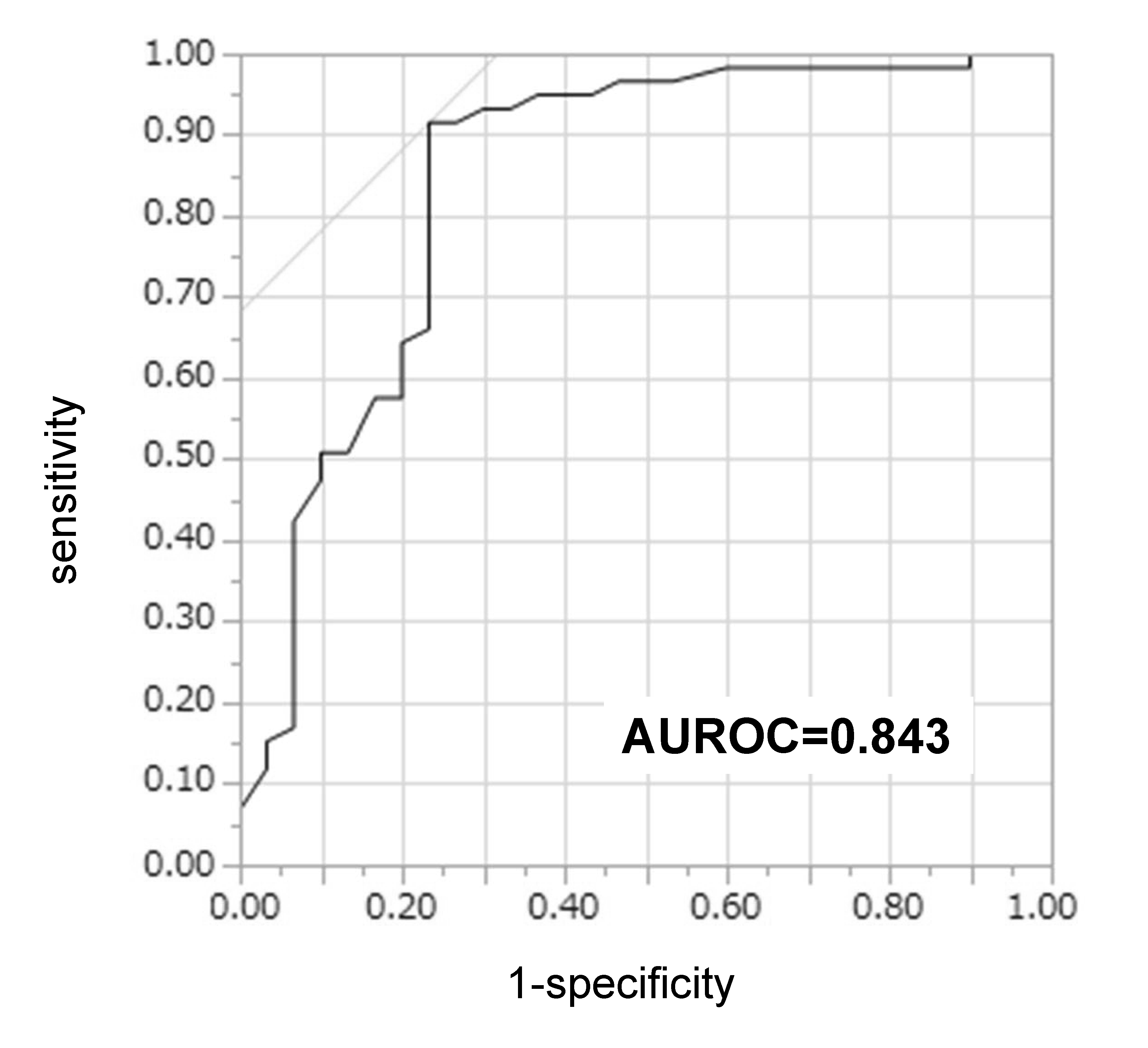

Supplement: Supplementary file 1 — Additional file 1. Fig. S1. Receiver operatorating characteristic curve of mean arterial pressure change by vasopressin loading to predict catecholamine index change after 6 h < 0. [file 13054_2023_4583_MOESM1_ESM.tiff]
