# Supplementary material for: The Vasopressin Loading for Refractory septic shock (VALOR) study: a prospective observational study
Source: Crit Care. 2023 Jul 21;27:294. doi: 10.1186/s13054-023-04583-7 (PMC10362561; doi:10.1186/s13054-023-04583-7)
Supplement: Supplementary file 2 — Additional file 2. Tables S1, S2 and S3. The sensitivity analysis of the baseline, outcomes and hemodynamics with the cut-off of 18 mmHg ΔMAP for responder/non-responder identification. [file 13054_2023_4583_MOESM2_ESM.docx]

**Additional file 2**

**Table S1. Differences in baseline characteristics between responders and non-responders to vasopressin loading with the cut-off of 18 mmHg mean arterial pressure change**

Data were expressed as a mean ± standard deviation for normally distributed continuous variables or median (interquartile range) for non-normally distributed continuous variables. Categorical variables were expressed as n (%).

MAP, mean arterial pressure; SOFA, sequential organ failure assessment; APACHE, acute physiology and chronic health evaluation; ACTH, adrenocorticotropic hormone

|  | **Responders** | **Non-responders** |  |
| --- | --- | --- | --- |
|  | **MAP change >18 mmHg** | **MAP change ≤18 mmHg** | **p** |
| **n** | **68** | **24** | **value** |
| age, years | 75.7±11.9 | 78.4±10.3 | 0.34 |
| male, n (%) | 46 (67.6) | 17 (70.8) | 0.92 |
| height, cm | 158.5±8.2 | 159.6±6.6 | 0.57 |
| body weight, kg | 55.4±13.7 | 52.8±8.8 | 0.40 |
| SOFA | 8 (6, 10) | 8.5 (5, 11) | 0.79 |
| APACHEII | 19 (14.8, 23.3) | 20 (13, 25.3) | 0.95 |
| mechanical ventilation, n (%) | 46 (67.6) | 17 (70.8) | 0.99 |
| blood purification, n (%) | 23 (33.8) | 13 (54.2) | 0.11 |
| extracorporeal membrane oxygenation, n (%) | 4 (5.9) | 2 (8.3) | 0.72 |
| atrial fibrillation, n (%) | 17 (25.0) | 5 (20.8) | 0.63 |
|  |  |  |  |
| vasopressin administration from noradrenaline start, hours | 5 (1.4, 15.5) | 1.75 (0.5, 4.4) | 0.017 |
| noradrenaline dose before loading, μg/kg/min | 0.3 (0.22, 0.5) | 0.4 (0.3, 0.6) | 0.31 |
| catecholamine index before loading | 30 (22, 50) | 43 (30, 60) | 0.31 |
| max lactate within 24 h of loading, mmol/L | 2.6 (1.6, 4.6) | 5.4 (3.0, 8.9) | 0.0064 |
|  |  |  |  |
| Vasopressin concentration, pg/ml | 5.8 (3.0, 10.6) | 7.7 (3.1, 28.0) | 0.45 |
| ACTH, pg/ml | 22.9 (9.7, 45.4) | 38.1 (22.3, 92.5) | 0.019 |
| cortisol, μg/dL | 21.9 (14.6, 34.0) | 27.0 (20.5, 41.2) | 0.085 |

**Table S2. Differences in outcomes between responders and non-responders to vasopressin loading** **with the cut-off of 18 mmHg mean arterial pressure change**

Data were expressed as a mean ± standard deviation for normally distributed continuous variables or median (interquartile range) for non-normally distributed continuous variables. Categorical variables were expressed as n (%).

MAP, mean arterial pressure; ICU, intensive care unit

|  | **Responder** | **Non-responder** |  |
| --- | --- | --- | --- |
|  | **MAP change >18 mmHg** | **MAP change ≤18 mmHg** | **p** |
| **n** | **68** | **24** | **value** |
| **Primary outcome** |  |  |  |
| catecholamine index change 6 h | -10 (-15, -5) | 1.5 (0, 28.8) | <0.0001 |
| **Secondary outcomes** |  |  |  |
| catecholamine index change 2 h | -2 (-10, 0) | 0 (0, 20) | 0.0004 |
| catecholamine index change 4 h | -10 (-10, 0) | 1.5 (0, 22.5) | <0.0001 |
|  |  |  |  |
| in-hospital death, n (%) | 25 (36.8) | 14 (58.3) | 0.094 |
| length of ICU stay, days | 7 (5, 9) | 7 (4.3, 11) | 0.90 |
| length of hospital stay, days | 21 (12, 47.5) | 20 (7.25, 42.3) | 0.61 |
| duration of mechanical ventilation, days | 5 (4, 9) | 7 (4.5, 11.5) | 0.22 |
| duration of blood purification, days | 5 (2, 19) | 6(5, 1) | 0.52 |
| Urine output 0-2 h, ml/h | 60 (18.8, 100) | 20 (0, 80) | 0.055 |
| Urine output 2-4 h, ml/h | 65 (20, 120) | 40 (6.3, 100) | 0.19 |
| Urine output 4-6 h, ml/h | 80 (35, 170) | 40 (6.3, 107.5) | 0.10 |
| Fluid volume IN 24h, ml | 2191 (1534, 3053) | 2886 (2026, 3483) | 0.033 |
| Fluid volume OUT 24h, ml | 1043 (674, 2308) | 843 (331, 1819) | 0.085 |
| Net IN-OUT balance, ml | 1007 (159, 1792) | 1668 (1154, 2736) | 0.0080 |
| pre lactate, mmol/L | 1.8 (1.3, 3.2) | 2.4 (1.4, 5.4) | 0.084 |
| post lactate, mmol/L | 1.6 (1.2, 2.5) | 2.8 (1.6, 5.45) | 0.0054 |
| lactate change 2 h, mmol/L | -0.2 (-0.8, 0.1) | 0.1 (-0.4, 0.7) | 0.0043 |
| pre pH | 7.40 (7.32, 7.48) | 7.38 (7.30, 7.45) | 0.46 |
| post pH | 7.40 (7.32, 7.45) | 7.41 (7.29, 7.44) | 0.69 |
| vasopressin administration time, hours | 40 (24.5, 61.3) | 45 (14.3, 85.9) | 0.73 |
| steroid use after vasopressin administration, n (%) | 14 (20.5) | 17 (58.3) | 0.0054 |
|  |  |  |  |
| digital ischemia, n (%) | 0 (0) | 2 (8.3) | 0.020 |
| mesenteric ischemia, n (%) | 1 (1.5) | 1 (4.2) | 0.48 |
| cardiac ischemia, n (%) | 1 (1.5) | 0 (0) | 0.43 |

**Table S3. Differences in hemodynamics between responders and non-responders to vasopressin loading with the cut-off of 18 mmHg mean arterial pressure change**

Data were expressed as a mean ± standard deviation for normally distributed continuous variables or median (interquartile range) for non-normally distributed continuous variables.

MAP, mean arterial pressure; SBP, systolic blood pressure, DBP, diastolic blood pressure; HR, heart rate; CO, cardiac output; SV, stroke volume; SVV, stroke volume variation, SVR, systemic vascular resistance; CVP, central venous pressure

|  | **Responder** | **Non-responder** |  |
| --- | --- | --- | --- |
|  | **MAP change >18 mmHg** | **MAP change ≤18 mmHg** | **p** |
| **n** | **68** | **24** | **value** |
| pre SBP mmHg | 105.8±19.8 | 105±22.5 | 0.87 |
| pre DBP mmHg | 51.6±10.2 | 49.4±11.6 | 0.39 |
| pre MAP mmHg | 69.4±12.1 | 67.0±14.9 | 0.24 |
| pre HR /min | 94.4±22.3 | 96.6±21.9 | 0.69 |
| pre CO l/min | 4.5 (3.6, 6.2) | 4 (3.6, 4.5) | 0.28 |
| pre SV ml | 49 (40, 58) | 41.5 (34, 63.5) | 0.27 |
| pre SVV % | 17 (12.5, 22) | 12 (8, 20) | 0.038 |
| pre dPmx | 860 (658, 1190) | 891 (505, 1377) | 0.93 |
| pre SVR dynes/sec/cm^5^ | 1165 (858, 1339) | 1195 (835, 1551) | 0.98 |
| pre CVP mmHg | 5 (5, 7) | 5 (4, 10.5) | 0.77 |
| post SBP mmHg | 157.5±25.3 | 121.7±27.0 | <0.0001 |
| post DBP mmHg | 75.4±12.5 | 59.5±14.6 | <0.0001 |
| post MAP mmHg | 105.1±12.2 | 78.0±15.8 | <0.0001 |
| post HR /min | 89.2±30.8 | 93.1±21.0 | 0.39 |
| post CO l/min | 4.6 (3.3, 5.7) | 3.7 (2.8, 4.4) | 0.070 |
| post SV ml | 52 (41, 68) | 42.5 (31.3, 54.5) | 0.034 |
| post SVV % | 12 (7, 17) | 12 (8, 20) | 0.58 |
| post dPmx | 1259 (1029, 1645) | 956 (538, 1504) | 0.046 |
| post SVR dynes/sec/cm^5^ | 1702 (1475, 2232) | 1445 (1057, 2131) | 0.078 |
| post CVP mmHg | 8.5 (5, 10) | 9.5 (5.5, 12) | 0.34 |
